# Multidimensional sleep health is associated with mild cognitive impairment in older adults: a cross-sectional study

**DOI:** 10.3389/fpubh.2026.1911714

**Published:** 2026-08-21

**Authors:** Yang Li, Xinxiu Dong, Hui Hu, Xiaotong Wang

**Affiliations:** 1 School of Nursing, Hubei University of Chinese Medicine, Wuhan, Hubei, China; 2 School of Medicine, Jianghan University, Wuhan, Hubei, China; 3 Hubei Shizhen Laboratory, Wuhan, Hubei, China

**Keywords:** cognitive function, cross-sectional study, mild cognitive impairment, older adults, sleep health

## Abstract

**Background:**

Mild cognitive impairment (MCI) is a transitional stage preceding dementia and may be amenable to early intervention. Although sleep problems are common among older adults and related to cognitive function, most studies have focused on single sleep characteristics rather than multidimensional sleep health.

**Methods:**

This cross-sectional study included 416 adults aged ≥ 60 years from three nursing homes and two communities in Wuhan, China. Sleep health was assessed using the Pittsburgh Sleep Quality Index (PSQI) and the Epworth Sleepiness Scale (ESS) to measure the five SATED sleep dimensions. Cognitive function was evaluated using the Montreal Cognitive Assessment Basic Version. Group comparisons and binary logistic regression analyses were used to examine associations between sleep health indicators and prevalent MCI.

**Results:**

Overall, 197 participants (47.35%) had MCI, and 294 (70.7%) had unfavorable sleep health in at least two SATED-based dimensions. The non-MCI group had a higher composite sleep health score than the MCI group (Z = −5.051, *p* < 0.001). In the fully adjusted model, each one-point increase in the composite sleep health score was associated with lower odds of prevalent MCI (OR = 0.692, 95% CI: 0.553–0.867, *p* = 0.001), and grouped analyses showed a significant decreasing trend in the odds of MCI across increasing sleep health score groups (P for trend = 0.004). Among individual sleep dimensions, sleep efficiency showed the most consistent association with MCI after full adjustment (OR = 0.510, 95% CI: 0.301–0.866, *p* = 0.013).

**Conclusion:**

Better multidimensional sleep health was associated with lower odds of prevalent MCI among older adults. Longitudinal studies are needed to confirm these findings.

## 1 Introduction

Cognitive impairment refers to declines in memory and other cognitive abilities and may arise from various neurological, psychiatric, vascular, and systemic conditions (1). Alzheimer’s disease (AD) is the most common cause of dementia, a major clinical outcome of progressive cognitive impairment. Mild cognitive impairment (MCI) is a transitional stage between the cognitive changes associated with normal aging and clinical dementia (2). It is estimated that 15%–28% of older adults with MCI progress to dementia within 1 year, and more than 70% do so within 5–10 years (3). However, another study (4) reported that 44% of older adults with MCI showed improvement in cognitive function after 1 year of cognitive management. Thus, for some individuals, MCI may represent a preclinical stage of dementia. Owing to its bidirectional transition and plasticity, MCI is considered an important window for early intervention. Therefore, identifying risk factors that can be modified is crucial to preventing dementia and its related burden.

As a basic physiological need of humans, sleep is closely associated with physical and mental health (5). Adequate sleep not only maintains physical vitality but also plays a crucial role in the regulation of cognitive function, memory consolidation, and emotional regulation (6). Good sleep quality facilitates the clearance of brain metabolites, supports brain function repair, and helps maintain homeostasis (7). In recent years, accumulating studies have explored the link between sleep and cognitive impairment. In a cross-sectional study conducted in China, insomnia and narcolepsy were associated with a higher likelihood of cognitive impairment among community residents, whereas obstructive sleep apnea (OSA) and restless legs syndrome were not significantly associated with cognitive impairment (8). In addition to sleep disorders, previous studies have linked both self-reported and objectively measured sleep characteristics to cognitive function. Specifically, long sleep latency (9), long wake after sleep onset (WASO) (10), poor sleep quality (11), and extreme sleep duration (too short or too long) (12, 13) are associated with cognitive decline.

Although prior studies have revealed the association between sleep and cognitive function from the perspectives of sleep disorders and individual sleep indicators, most studies have focused on a single sleep problem or parameter. Few studies have systematically investigated this association from an integrated, multidimensional perspective of positive sleep health. In 2014, Buysse (14) first proposed the concept of “sleep health,” defining it as “multiple dimensions of sleep that adapt to individual, social, and environmental needs and promote physical and mental health.” He also developed the SATED scale to assess five sleep dimensions: satisfaction, alertness, sleep timing, sleep efficiency, and sleep duration (14). To date, studies on sleep health have mainly focused on its relationships with metabolic diseases and mental disorders (15), while evidence regarding cognitive function remains limited. A 10-year cohort study (16) demonstrated that the degree of sleep health impairment in older men is positively correlated with the risk of cognitive decline. Calfee et al. (17) and Boeve et al. (9) also reported that poorer sleep health was associated with worse cognitive-related outcomes across different age groups. However, existing relevant evidence is predominantly derived from Western populations, and empirical data among Chinese older adults remains limited. Therefore, this study aimed to examine the associations of multidimensional SATED-based sleep health, including individual sleep dimensions and a composite sleep health score, with prevalent MCI among older adults in China.

## 2 Materials and methods

### 2.1 Population

Participants were recruited from three nursing homes and two communities in Wuhan using a convenience sampling method. The sample size was calculated using the formula of [Z^2^P(1-P)]/d^2^, with a significance level set at 0.05 and CI of 95% (18). The prevalence of MCI among older adults in Chinese nursing homes was set at 0.54 (19) with an absolute precision of 0.05. After accounting for a 10% invalid sample size, the final calculated sample size was 425 participants. Inclusion criteria: (1) Age ≥ 60 years; (2) having lived in the selected nursing homes or communities for at least 6 months; and (3) having sufficient communication and comprehension abilities to complete the questionnaire. Exclusion criteria: (1) a diagnosis of dementia, such as Alzheimer’s disease or vascular dementia, or other neurological diseases that may cause brain dysfunction, as diagnosed by a medical institution; (2) severe visual or hearing impairment; (3) comorbid psychiatric or neurological disorders that result in the inability to communicate normally. Prior to the interviews, all participants provided informed consent. The study was approved by the Ethics Committee of Jianghan University (Approved No. of ethic committee: JHDXKJLL2025-261).

### 2.2 Measures

#### 2.2.1 Sleep health assessment

Sleep health was assessed based on the five sleep health dimensions of Buysse’s SATED framework: satisfaction/subjective sleep quality, alertness, timing, efficiency, and duration (14). In this study, these dimensions were measured using standardized self-report sleep scales, including the Pittsburgh Sleep Quality Index (PSQI) and the Epworth Sleepiness Scale (ESS). Subjective sleep quality was assessed using PSQI Item 6. Participants with a score of 0 or 1 were classified as having favorable subjective sleep quality, whereas those with a score of 2 or 3 were classified as having poor subjective sleep quality. This variable reflected subjective sleep quality only and was not intended to represent global sleep quality or sleep disturbance as defined by the PSQI total score. Sleep efficiency was calculated using PSQI Items 1, 3, and 4. Sleep timing was derived from PSQI Items 1 and 3, whereas sleep duration was based on PSQI Item 4. According to previous research (20), the daytime alertness dimension was operationalized using the Epworth Sleepiness Scale (ESS), which assesses the tendency toward daytime sleepiness. An ESS score ≤ 10 was defined as the absence of excessive daytime sleepiness and was used as a proxy for favorable status in the daytime alertness dimension. Each sleep dimension was dichotomized as favorable sleep health, scored as 1, or poor sleep health, scored as 0, according to cut-off values used in previous studies (Table 1) (21–25). The composite sleep health score was calculated by summing the scores of the five dimensions, yielding a total score ranging from 0 to 5, which represented the number of sleep health dimensions classified as good. Higher scores indicated better overall sleep health (26).

**Table 1:** Sleep health dimensions and cut points used to construct the sleep health composite score.

Dimension	Definition (14)	Assessment/Item	Cut point
Subjective sleep quality/satisfaction (21)	The subjective assessment of “good” or “poor” sleep	Self-rated sleep quality score (from PSQI Item 6)	1: Item 6 of the PSQI had a score of 0 or 1. 0: Item 6 of the PSQI had a score of 2 or 3.
Alertness (22)	The ability to maintain attentive wakefulness	Using the Epworth Sleepiness Scale (ESS)	1: ESS ≤ 10 0: ESS > 10
Sleep timing (23)	The placement of sleep within the 24-h day.	It was expressed as the sleep midpoint, calculated by the formula: Bedtime + (Wake-up Time − Bedtime) / 2. Two items from the PSQI (Items 1, 3) were used to calculate the sleep midpoint.	1: 2:00–4:00 a.m. 0: < 2:00 a.m. / > 4:00 a.m.
Sleep efficiency (24)	The proportion of time in bed spent asleep.	Three items from the PSQI (Items 1, 3, and 4) were used to calculate sleep efficiency.	1: ≥ 85% 0: < 85%
Sleep duration (25)	The total amount of sleep obtained per 24 h.	PSQI (Items 4): “How many hours do you actually sleep each night?”	1: 7-9 h 0: < 7 h or > 9 h

PSQI, Pittsburgh Sleep Quality Index.

#### 2.2.2 Cognitive function assessment

The Montreal Cognitive Assessment (MoCA) is a widely used screening tool for cognitive function among older adults. Previous studies have shown that the MoCA has high sensitivity (80%–100%) and moderate specificity (50%–76%) for identifying MCI (27). In addition, the MoCA is superior to the Mini-Mental State Examination (MMSE) in distinguishing individuals with normal cognition from those with MCI and has received a Grade A recommendation (28). Based on the expert consensus on neuropsychological assessment in older adults, the Montreal Cognitive Assessment-Basic (MoCA-B) was used to evaluate global cognitive function in this study (28). The Chinese version of the MoCA-B is specifically designed for older adults with low educational attainment and can reduce ceiling effects among highly educated individuals (29). The MoCA-B consists of eight cognitive subdomains: executive function, language, orientation, calculation, conceptual thinking, memory, visual perception and attention (www.mocatest.org, visit Basic section). The total score is 30, with higher scores indicating better cognitive function. Education-stratified cut-off values were adopted for MCI screening: ≤19 points for participants with ≤6 years of education, ≤22 points for those with 7–12 years of education, and ≤24 points for those with >12 years of education (29). The Chinese version of the MoCA-B has demonstrated good internal consistency, with a Cronbach’s *α* coefficient of 0.807, and these education-specific cut-offs have shown optimal sensitivity and specificity across educational subgroups (29). Before data collection, all investigators received standardized training on questionnaire administration, cognitive assessment procedures, scoring criteria, and quality control. Cognitive assessments were conducted by trained investigators, with the participation and guidance of qualified physicians. Participants whose MoCA-B scores were below the corresponding education-specific cut-offs underwent further cognitive assessment, including the Activities of Daily Living (ADL) scale and the Clinical Dementia Rating (CDR) scale. Finally, MCI diagnosis was determined according to Petersen’s criteria (2), published clinical guidelines and expert consensus (30), based on the following criteria: (1) subjective memory complaints; (2) MoCA-B score lower than the education-specific cut-off value; (3) normal or mild impairment in activities of daily living (ADL score < 16); (4) Clinical Dementia Rating (CDR) score of 0.5.

#### 2.2.3 Covariates

Candidate covariates were selected based on previous literature and clinical relevance and included sociodemographic characteristics, lifestyle factors, and health-related variables. Sociodemographic variables included age, sex, educational level, marital status, residential status, and monthly income. Lifestyle-related variables included smoking status, alcohol consumption, tea consumption, physical exercise, and participation in social activities. Health-related variables included the number of chronic diseases and mental health status. Mental health status was assessed using the Kessler Psychological Distress Scale (K6), a six-item instrument designed to measure nonspecific psychological distress (31). The K6 assesses how often respondents experienced the following symptoms during a specified period: feeling nervous, depressed, restless or fidgety, hopeless, that everything was an effort, and worthless. Each item is rated on a 5-point Likert scale, and the total score ranges from 0 to 24, with higher scores indicating greater psychological distress. In this study, a total K6 score >12 was used to indicate a high risk of psychological distress. Other covariates were collected using a researcher-developed questionnaire.

### 2.3 Statistical analysis

A total of 430 participants were initially enrolled. Fourteen participants (3.26%) were excluded because of incomplete questionnaire data, leaving 416 participants for the final analysis. The normality of continuous variables was assessed before statistical analysis. Normally distributed continuous variables are presented as the mean ± standard deviation (SD), whereas non-normally distributed variables are presented as the median and interquartile range [M(P25, P75)]. Categorical variables are presented as frequencies and percentages. For comparisons between participants with and without MCI, independent-samples *t* tests were used for normally distributed continuous variables, and Mann–Whitney U tests were used for non-normally distributed continuous variables. The chi-square test was used for categorical variables, and the Wilcoxon rank-sum test was used for ordinal categorical variables, as appropriate. All statistical tests were two-sided, and a *p* value < 0.05 was considered statistically significant.

**Figure 1:**
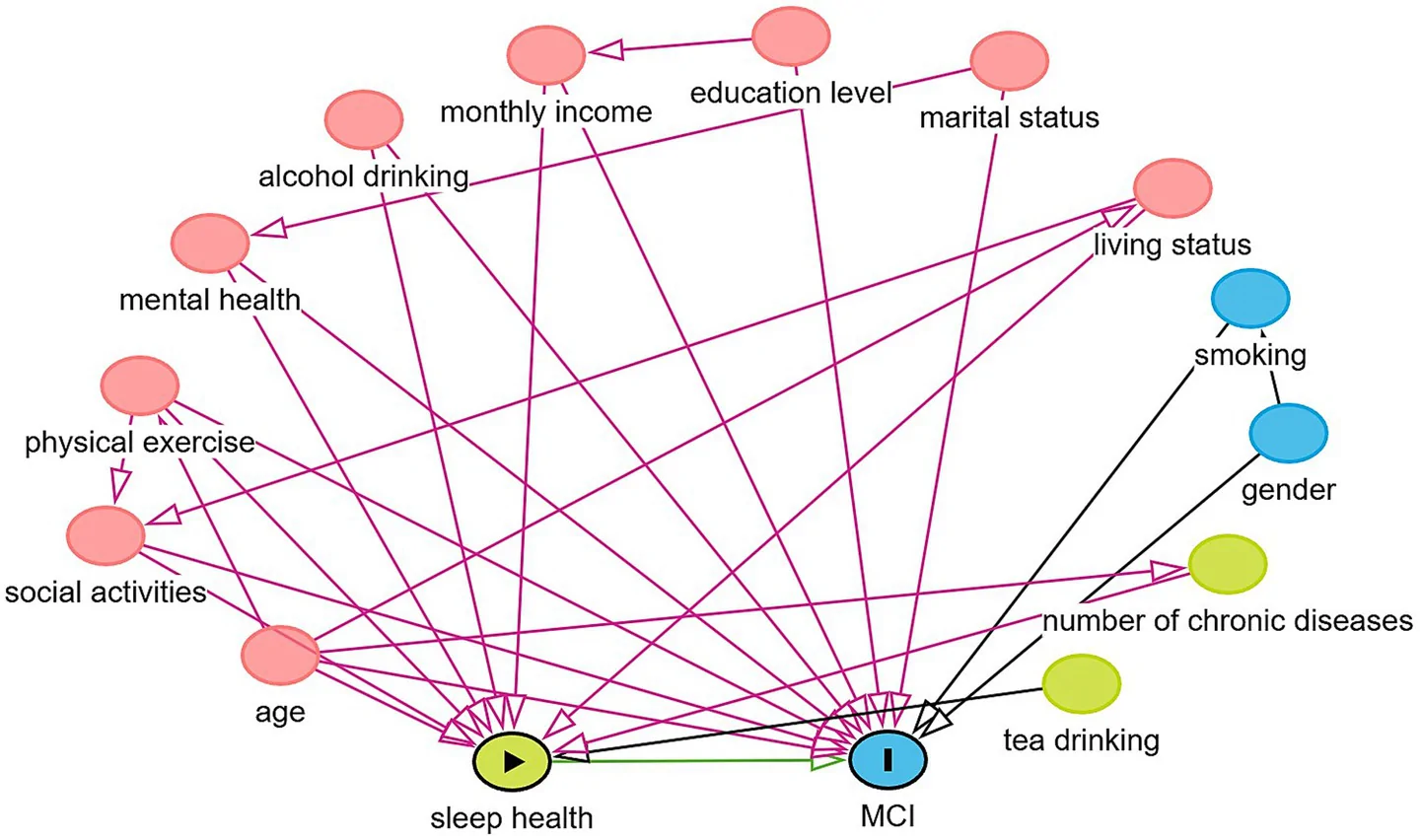
Directed acyclic graph of the association between sleep health and mild cognitive impairment. The directed acyclic graph illustrates the hypothesized causal relationships among sleep health, mild cognitive impairment, and covariates. The graph was constructed using DAGitty software.

A directed acyclic graph (DAG) was constructed using DAGitty software to guide covariate selection (32). First, sleep health was specified as the exposure and MCI as the outcome. Second, candidate covariates were identified based on previous literature and clinical relevance, including sociodemographic characteristics, lifestyle factors, and health-related variables. Third, hypothesized causal pathways among these variables were specified according to prior evidence and theoretical assumptions. In the DAG, arrows represent assumed causal directions between variables. Fourth, the adjustment-set function in DAGitty was used to identify the minimum sufficient adjustment set required to block non-causal backdoor paths between sleep health and MCI. The DAG-derived minimum sufficient adjustment set included age, monthly income, physical exercise, alcohol consumption, social activities, and mental health status. These variables were included in Model 2, as illustrated in Figure 1.

Binary logistic regression models were used to examine the associations of individual sleep health dimensions and the composite sleep health score with MCI. The outcome variable was MCI status, coded as 1 for participants diagnosed with MCI and 0 for those without MCI. Odds ratios (ORs) and 95% confidence intervals (CIs) were calculated to quantify the associations between sleep health variables and MCI. First, the five individual sleep health dimensions were simultaneously entered into the regression models to evaluate their independent associations with MCI. Second, the composite sleep health score was examined separately to assess the association between overall multidimensional sleep health and MCI. The individual sleep health dimensions and the composite sleep health score were not included in the same model because the composite score was derived from the five dimensions. Three models were constructed for both sets of analyses. Model 1 was unadjusted. Model 2 was adjusted for the minimum sufficient adjustment set identified using a directed acyclic graph (DAG), including age, monthly income, alcohol consumption, physical exercise, social activities, and mental health status. Based on Model 2, Model 3 further adjusted for educational level, marital status, sex, and smoking status, which differed significantly between the MCI and non-MCI groups and were considered clinically relevant, to reduce possible residual confounding and assess the robustness of the associations. For the composite sleep health score, we also calculated P for trend using binary logistic regression models to further evaluate the dose–response trend. The composite sleep health score was categorized into four groups: 0–1, 2, 3, and 4–5. The lowest score group (0–1) was used as the reference group. Trend tests were conducted in the unadjusted model (Model 1), the DAG-adjusted model (Model 2), and the fully adjusted model (Model 3). In addition, given previous evidence of sex differences in sleep characteristics and cognitive aging (20), we conducted exploratory sex-stratified logistic regression analyses to assess whether the association between the composite sleep health score and MCI was consistent across male and female participants. These models were adjusted for the same covariates as the fully adjusted model, except for sex. All statistical tests were two-tailed, and a *p* < 0.05 was considered statistically significant. Statistical analyses were performed using IBM SPSS Statistics (version 27.0).

## 3 Results

### 3.1 Characteristics of the participants

This study conducted face-to-face questionnaire surveys among 430 participants from three nursing homes and two communities in Wuhan from March to August 2025. After eliminating 14 participants because of invalid or incomplete questionnaire data, 416 participants were included in the final analysis, yielding a valid response rate of 96.74%. The characteristics of the participants are presented in Table 2. The mean age of participants was 70.55 years (SD = 7.58), and the mean body mass index (BMI) was 23.36 kg/m^2^ (SD = 2.97). According to the MoCA-B-based MCI diagnostic criteria described above, 197 participants (47.35%) were diagnosed with MCI. Baseline demographic and clinical characteristics were further compared between the MCI group and non-MCI group. The results revealed that age, sex, marital status, education level, monthly income, physical exercise, social activities, alcohol consumption, smoking status and mental health status were significantly different between the two groups (*p* < 0.05).

**Table 2:** Participant characteristics and group comparison for MCI (*N* = 416).

Variables	Total (*N* = 416)	MCI		*p*
		No (*n* = 219)	Yes (*n* = 197)	
Age (years)	70.5(7.6)	68.7 (6.3)	72.5 (8.3)	<0.001
BMI (kg/m^2^)	23.4 (2.9)	23.4 (2.8)	23.3 (3.1)	0.524
Sex				0.003
Male	171 (41.1%)	105 (47.9%)	66 (33.5%)	
Female	245 (58.9%)	114 (52.1%)	131 (66.5%)	
Marital status				0.001
Married	237 (57.0%)	141 (64.4%)	96 (48.7%)	
Single/divorced/separated	179 (43.0%)	78 (35.6%)	101 (51.3%)	
Residential status				0.343
Living alone	23 (5.5%)	14 (6.4%)	9 (4.6%)	
With spouse/children	114 (27.4%)	54 (24.7%)	60 (30.5%)	
Nursing home	279 (67.1%)	151 (68.9%)	128 (65.0%)	
Education level				<0.001
Illiteracy/Primary school	32 (7.7%)	7 (3.2%)	25 (12.7%)	
Junior and high school	254 (61.1%)	149 (68.0%)	105 (53.3%)	
University or above	130 (31.3%)	63 (28.8%)	67 (34.0%)	
Monthly income (RMB)				<0.001
<2,000	146 (35.1%)	55 (25.1%)	91 (46.2%)	
2,000–4,000	154 (37.0%)	76 (34.7%)	78 (39.6%)	
>4,000	116 (27.9%)	88 (40.2%)	28 (14.2%)	
Physical exercise				0.018
Never	120 (28.8%)	58 (26.5%)	62 (31.5%)	
1–3 times/week	242 (58.2%)	123 (56.2%)	119 (60.4%)	
>3 times/week	54 (13.0%)	38 (17.4%)	16 (8.1%)	
Social activities				0.023
Never	73 (17.5%)	32 (14.6%)	41 (20.8%)	
1–3 times/week	282 (67.8%)	146 (66.7%)	136 (69.0%)	
>3 times/week	61 (14.7%)	41 (18.7%)	20 (10.2%)	
Smoking				<0.001
Yes	141 (33.9%)	91 (41.6%)	50 (25.4%)	
No	275 (66.1%)	128 (58.4%)	147 (74.6%)	
Tea drinking				0.213
Yes	181 (43.5%)	89 (40.6%)	92 (46.7%)	
No	235 (56.5%)	130 (59.4%)	105 (53.3%)	
Alcohol consumption				0.020
Yes	203 (48.8%)	95 (43.4%)	108 (54.8%)	
No	213 (51.2%)	124 (56.6%)	89 (45.2%)	
Number of chronic diseases				0.857
0	89 (21.4%)	49 (22.4%)	40 (20.3%)	
1–2	264 (63.5%)	138 (63.0%)	126 (64.0%)	
>3	63 (15.1%)	32 (14.6%)	31 (15.1%)	
Kessler psychological distress scale score	5.7 (3.7)	4.8 (3.5)	6.8 (3.7)	<0.001

BMI, body mass index; data are presented as mean (SD) or *n* (%).

### 3.2 Sleep health of the participants

Table 3 presents the sleep health status of all enrolled participants. Among 416 participants, the mean composite sleep health score was 2.77 (SD = 1.21). The most prevalent composite sleep health scores were 2 points (25.2%) and 3 points (28.4%). A total of 294 participants (70.7%) had unfavorable sleep health in at least two SATED-based dimensions. Across the five individual sleep health dimensions, the median sleep midpoint was 2:00 a.m. Regarding unfavorable sleep health status, 146 participants (35.1%) reported poor subjective sleep quality, 265 (63.7%) had low sleep efficiency, and 246 (59.1%) had inappropriate sleep duration, defined as either short or long sleep duration. In the daytime alertness dimension, 65 participants (15.6%) had unfavorable status, defined as excessive daytime sleepiness based on an ESS score >10.

**Table 3:** Sleep health of the participants (*N* = 416).

Variables	Statistical value	Range
Composite sleep health score	2.77 (1.21)	0–5
0	4 (1.0%)	
1	67 (16.1%)	
2	105 (25.2%)	
3	118 (28.4%)	
4	87 (20.9%)	
5	35 (8.4%)	
*Individual sleep dimension*		
Sleep timing	2:00 (1:30, 02:15)	0:00–5:01
<2:00 a.m. or >4:00 a.m.	204 (49.0%)	
Subjective sleep quality	1.00 (1.00, 2.00)	0–3
PSQI item 6 > 1	146 (35.1%)	
Sleep efficiency (%)	78.16 (66.67, 87.50)	35–98
<85%	265 (63.7%)	
Alertness	5.00 (2.00, 7.00)	0–20
ESS > 10	65 (15.6%)	
Sleep duration (h)	6.50 (6.00, 7.50)	4–10
<7 h or >9 h	246 (59.1%)	

PSQI, Pittsburgh Sleep Quality Index; ESS, Epworth Sleepiness Scale. Data are presented as mean (SD), median (P25, P75), or *n* (%), as appropriate.

### 3.3 Association between sleep health and cognitive function

Table 4 compares the composite sleep health score and corresponding five dimensions between the MCI group and non-MCI group. Compared with the non-MCI group, the MCI group had an earlier sleep midpoint, worse subjective sleep quality, lower sleep efficiency and significantly lower composite sleep health score. By contrast, no significant between-group differences were detected in daytime alertness or sleep duration.

**Table 4:** Comparison of sleep health between the MCI and non-MCI groups.

Variables	Non-MCI	MCI	*Z*	*p*
Sleep timing	02:00 (01:38, 02:30)	01:45 (01:15, 02:08)	−4.603	<0.001
Subjective sleep quality	1.00 (1.00, 2.00)	1.00 (1.00, 2.00)	4.964	<0.001
Sleep efficiency (%)	81.00 (70.00, 88.89)	75.00 (64.29, 85.16)	−3.734	<0.001
Alertness	4.00 (2.00, 7.00)	5.00 (3.00, 7.50)	1.397	0.162
Sleep duration (h)	7.00 (6.00, 7.50)	6.20 (5.50, 8.00)	−1.676	0.094
Composite sleep health score	3.00 (2.00, 4.00)	2.00 (2.00, 3.00)	−5.051	< 0.001

Data are presented as median (P25, P75). *Z* values were obtained from the Mann–Whitney U test.

Binary logistic regression models were conducted to examine the association of individual sleep health dimensions with MCI (Table 5). In Model 1, which included the five sleep health dimensions without adjustment for external covariates, favorable subjective sleep quality (OR = 0.471, 95% CI: 0.305–0.727), high sleep efficiency (OR = 0.482, 95% CI: 0.311–0.747), and favorable sleep timing (OR = 0.601, 95% CI: 0.399–0.906) were significantly associated with lower odds of prevalent MCI. After adjustment for age, monthly income, alcohol consumption, physical exercise, social activities, and mental health status in Model 2, favorable subjective sleep quality (OR = 0.600, 95% CI: 0.362–0.994) and high sleep efficiency (OR = 0.483, 95% CI: 0.292–0.801) remained significantly associated with lower odds of prevalent MCI. In Model 3, only sleep efficiency remained significantly associated with MCI. Compared with participants with poor sleep efficiency, those with favorable sleep efficiency had significantly lower odds of MCI (OR = 0.510, 95% CI: 0.301–0.866). In contrast, daytime alertness, subjective sleep quality, sleep duration, and sleep timing were not significantly associated with MCI after full adjustment.

**Table 5:** Binary logistic regression models for the associations between sleep health dimensions and MCI (*N* = 416).

Model	B	*SE*	*p*	OR(95% CI)
Model 1				
Alertness^1^	0.134	0.284	0.637	1.143 (0.656–1.994)
Subjective sleep quality ^2^	−0.753	0.222	<0.001	0.471 (0.305–0.727)
Sleep duration^3^	−0.026	0.221	0.905	0.974 (0.632–1.502)
Sleep efficiency^4^	−0.731	0.224	0.001	0.482 (0.311–0.747)
Sleep timing^5^	−0.509	0.209	0.015	0.601 (0.399–0.906)
Model 2				
Alertness^1^	0.232	0.324	0.473	1.261 (0.669–2.380)
Subjective sleep quality^2^	−0.511	0.258	0.048	0.600 (0.362–0.994)
Sleep duration^3^	−0.288	0.265	0.278	0.750 (0.446–1.260)
Sleep efficiency^4^	−0.728	0.258	0.005	0.483 (0.292–0.801)
Sleep timing^5^	−0.402	0.236	0.089	0.669 (0.421–1.063)
Model 3				
Alertness^1^	0.146	0.333	0.661	1.157 (0.602–2.224)
Subjective sleep quality^2^	−0.445	0.289	0.123	0.641 (0.364–1.129)
Sleep duration^3^	−0.321	0.274	0.241	0.725 (0.424–1.241)
Sleep efficiency^4^	−0.673	0.270	0.013	0.510 (0.301–0.866)
Sleep timing^5^	−0.282	0.244	0.248	0.754 (0.467–1.217)

^1^Reference group = Poor Alertness; ^2^Reference group = Poor subjective sleep quality; ^3^Reference group = Sleep duration <7 h or >9 h; ^4^Reference group = Poor sleep efficiency; ^5^Reference group = Poor sleep timing; OR, Odds Ratio; CI, Confidence Interval.

Model 1 was unadjusted.

Model 2 was adjusted for age, monthly income, alcohol consumption, physical exercise, social activities, and mental health status.

Model 3 was additionally adjusted for educational level, marital status, sex, and smoking status based on Model 2.

Table 6 presents the association between the composite sleep health score and MCI. First, the composite sleep health score was entered into the binary logistic regression model as a continuous variable. In the fully adjusted model, a higher composite sleep health score was significantly associated with lower odds of prevalent MCI (OR = 0.692, 95% CI: 0.553–0.867, *p* = 0.001). The composite sleep health score was further categorized into four groups: 0–1, 2, 3, and 4–5. Compared with participants in the lowest score group (0–1), those in the highest score group (4, 5) had significantly lower odds of MCI in the unadjusted model (Model 1: OR = 0.295, 95% CI: 0.160–0.543, *p* < 0.001), the DAG-adjusted model (Model 2: OR = 0.301, 95% CI: 0.144–0.630, *p* = 0.001), and the fully adjusted model (Model 3: OR = 0.363, 95% CI: 0.164–0.804, *p* = 0.012). Significant decreasing trends in the odds of MCI were observed across increasing sleep health score groups in all models (*P* for trend < 0.001 in Models 1 and 2; *P* for trend = 0.004 in Model 3).

**Table 6:** Associations between the composite sleep health score and MCI using binary logistic regression models (*N* = 416).

Variables	Model 1	*p*	Model 2	*p*	Model 3	*p*
	OR (95% CI)		OR (95% CI)		OR (95% CI)	
Composite sleep health score	0.647 (0.546–0.767)	<0.001	0.653 (0.530–0.804)	<0.001	0.692 (0.553–0.867)	0.001
Composite sleep health score group						
0–1	1.00 (reference)		1.00 (reference)		1.00 (reference)	—
2	0.939 (0.508–1.736)	0.841	0.992 (0.493–1.999)	0.983	0.965 (0.460–2.022)	0.924
3	0.549 (0.302–0.999)	0.050	0.632 (0.312–1.283)	0.204	0.684 (0.321–1.458)	0.325
4–5	0.295 (0.160–0.543)	<0.001	0.301 (0.144–0.630)	0.001	0.363 (0.164–0.804)	0.012
*P* for trend		<0.001		<0.001		0.004

OR, odds ratio; CI, confidence interval.

Model 1 was unadjusted.

Model 2 was adjusted for age, monthly income, alcohol consumption, physical exercise, social activities, and mental health status.

Model 3 was additionally adjusted for educational level, marital status, sex, and smoking status based on Model 2.

We further conducted exploratory sex-stratified logistic regression analyses to examine whether the association between the composite sleep health score and MCI was consistent across male and female participants. In the fully adjusted model, higher composite sleep health scores were significantly associated with lower odds of prevalent MCI in both male participants (OR = 0.634, 95% CI: 0.432–0.929) and female participants (OR = 0.713, 95% CI: 0.533–0.954). These exploratory findings are presented in Supplementary Table S1.

## 4 Discussion

This cross-sectional study examined the association between multidimensional sleep health and prevalent MCI among older adults. We assessed sleep health across five dimensions: subjective sleep quality, daytime alertness, sleep timing, sleep efficiency, and sleep duration. The mean composite sleep health score was 2.77 (SD = 1.21), and 70.7% of participants had unfavorable sleep health in at least two dimensions. Poor sleep efficiency and abnormal sleep duration were the two most common unfavorable sleep health dimensions. After adjustment for potential confounders, a higher composite sleep health score was associated with lower odds of prevalent MCI, suggesting that overall multidimensional sleep health may be related to cognitive status in later life. After adjustment for all covariates, sleep efficiency was the only individual dimension that remained significantly associated with MCI. The attenuation of the associations for sleep duration and subjective sleep quality after covariate adjustment may reflect confounding by demographic, lifestyle, or health-related factors, as well as overlap among sleep dimensions. However, these findings do not necessarily indicate that other sleep dimensions are irrelevant; rather, their contributions may be partly shared with other sleep characteristics or better captured by the composite sleep health score. Given the cross-sectional design, causal inference cannot be made. Overall, our findings highlight the importance of assessing sleep health from a multidimensional perspective rather than relying on single sleep indicators.

The association between the composite sleep health score and prevalent MCI supports the value of assessing sleep health as a multidimensional construct. Traditional sleep research has often focused on isolated indicators (1), such as sleep duration or subjective sleep quality, or on global measures of sleep disturbance, which may not fully capture an individual’s overall sleep health profile. Unlike traditional sleep measures such as the PSQI, which primarily assess subjective sleep quality and sleep-related disturbances, the SATED framework conceptualizes sleep as a positive and multidimensional health construct. This framework provides a broader profile of sleep health and allows the cumulative burden of unfavorable sleep characteristics to be considered. In the present study, PSQI- and ESS-derived indicators were reorganized within the SATED framework to construct a composite sleep health score. This approach enabled us to evaluate overall sleep health rather than relying on any single sleep indicator or global sleep quality score alone. The composite sleep health score showed a robust association with prevalent MCI, whereas associations for individual sleep dimensions were less consistent after adjusting for the covariates. These findings suggest that multidimensional sleep health may better capture sleep-related vulnerability to cognitive impairment than isolated sleep characteristics. Our results are consistent with those reported by Calfee et al. (17), who found that better multidimensional sleep health was associated with better cognitive performance, further supporting the relevance of the SATED framework in cognitive aging research.

Among all sleep indicators, sleep efficiency was the only individual sleep dimension that remained significantly associated with MCI after full adjustment. After adjustment for confounding covariates, older adults with high sleep efficiency had a 49.0% lower odds of prevalent MCI relative to those with poor sleep efficiency, which was consistent with findings from previous studies (17, 33). As a core component of overall sleep health, sleep efficiency may serve as an important correlate of the cross-sectional association between sleep health and cognitive function. Beyond its influence on global cognitive performance, poor sleep efficiency is also independently associated with impairments in memory and language functions (34). Potential biological explanations remain speculative. Inefficient sleep may be related to reduced restorative sleep processes, poorer memory consolidation, and impaired metabolic clearance during sleep (35, 36), all of which have been implicated in cognitive aging. However, these pathways were not directly measured in our study and should be examined in future longitudinal and mechanistic research.

Except for sleep efficiency, the associations of subjective sleep quality, sleep duration, sleep timing, and alertness with MCI became non-significant after covariate adjustment. This attenuation may be partly explained by confounding factors and interrelations among sleep dimensions. Sociodemographic and lifestyle factors, such as age, education, and health behaviors, are closely related to both sleep and cognitive function (37), and adjustment for these factors may have weakened the crude associations. Moreover, individual sleep dimensions are not independent but may jointly reflect overall sleep health (37). For example, Luo et al. (38) reported that sleep quality modified the associations of nocturnal sleep duration and daytime napping with MCI, suggesting that single sleep indicators may not consistently capture sleep-related cognitive vulnerability.

Measurement and classification issues may also have contributed to these inconsistent findings. Subjective sleep quality was assessed using a single PSQI item, which may not fully capture the complexity of perceived sleep experience and may be influenced by mood, physical discomfort, and psychological stress (39). Wallace et al. (40) showed that qualitative sleep domains, particularly sleep quality and alertness, are more difficult to define and harmonize across cohorts than quantitative sleep domains. For sleep timing, the favorable sleep midpoint defined in this study may not fully fit the sleep–wake patterns of institutionalized older adults, and PSQI-derived sleep timing may not adequately capture circadian rhythm stability, weekday–weekend differences, or daytime naps. In addition, because the association between sleep duration and cognition may be nonlinear or U-shaped (12, 20, 37, 39), combining short and long sleep duration into one “inappropriate sleep duration” category may have obscured their distinct associations with MCI. These considerations further support the use of a multidimensional sleep health score rather than reliance on individual sleep indicators alone.

The present study showed that each one-point increase in the composite sleep health score, corresponding to one additional favorable sleep health dimension, was associated with a 30.8% lower odds of prevalent MCI. In addition, the grouped analysis further supported this association, showing a significant decreasing trend in the odds of MCI across increasing composite sleep health score groups after full adjustment (P for trend = 0.004). Specifically, participants in the highest score group (4–5) had significantly lower odds of MCI than those in the lowest score group (0–1), suggesting a graded association between better multidimensional sleep health and lower likelihood of MCI. Wu et al. (37) similarly showed that the co-occurrence of multiple unfavorable sleep characteristics was associated with lower MMSE scores, suggesting that multiple sleep dimensions may influence cognitive function independently, additively, or synergistically. Smevik et al. (33) reported that poorer sleep health was associated with altered brain activation during cognitive control processing. These findings suggest that co-existing impairments in multiple sleep health dimensions may be related to broader disturbances in cognitive-control-related neural systems. However, because our study did not assess neuroimaging markers or formally examine interactions among sleep dimensions, these mechanisms remain speculative. Accordingly, future sleep management for older adults at risk of MCI may benefit from comprehensive improvements across multiple sleep health dimensions. A multidomain sleep health management framework could help guide more precise and effective non-pharmacological strategies for supporting cognitive health in later life.

In exploratory sex-stratified analyses, the inverse association between multidimensional sleep health and prevalent MCI was observed in both male and female participants after covariate adjustment. Although the association appeared slightly stronger among male participants, this finding should be interpreted cautiously because of the limited sample size within sex strata. Future studies should further examine potential sex-specific pathways linking sleep health to cognitive aging.

Several limitations should be noted in this study. First, this study adopted a cross-sectional design, which failed to support causal inference between multidimensional sleep health and MCI prevalence. Meanwhile, reverse causality was not excluded: prodromal brain degenerative changes prior to MCI diagnosis may disrupt central sleep regulatory centers and further deteriorate sleep profiles, which needs further verification via longitudinal cohort design. Second, all sleep health and mental health indicators were collected through subjective questionnaire scales, which inevitably caused recall bias and reporting bias; the absence of objective sleep monitoring indicators may underestimate the actual association magnitude. Third, convenience sampling was adopted, and all participants were recruited from communities and nursing homes in Wuhan only, which limited the national representativeness and population generalizability of the research findings. Fourth, this study failed to collect objective brain imaging and biological biomarker data, which restricted further exploration of potential mediating and neural mechanisms. In the future, multicenter, long-term prospective cohort studies combining subjective questionnaires and objective wearable sleep devices, neuroimaging and biological indicators are required to clarify the causal and mechanistic links between sleep health and cognitive impairment in older adults.

## 5 Conclusion

This cross-sectional study examined the associations between multidimensional SATED-based sleep health and prevalent MCI among older adults in Wuhan. Better overall multidimensional sleep health, operationalized using a SATED-based composite score, was associated with lower odds of prevalent MCI. The grouped analysis further showed a significant decreasing trend in the odds of MCI across increasing composite sleep health score groups, supporting a graded association between better multidimensional sleep health and lower likelihood of MCI. Among individual sleep dimensions, sleep efficiency showed the most consistent association with MCI after full adjustment. Prospective studies incorporating objective sleep measures and neuroimaging data are needed to confirm these findings and clarify the underlying mechanisms.

## Data Availability

The original contributions presented in the study are included in the article/Supplementary material, further inquiries can be directed to the corresponding author.
