# Supplementary material for: Multidimensional sleep health is associated with mild cognitive impairment in older adults: a cross-sectional study
Source: Front Public Health. 2026 Aug 21;14:1911714. doi: 10.3389/fpubh.2026.1911714 (PMC13541560; doi:10.3389/fpubh.2026.1911714)
Supplement: Supplementary file 1 [file Table_1.DOCX]

Supplementary Table S1. Sex-stratified associations between composite sleep health score and MCI

| **Sex** | **Total (n)** | **MCI n (%)** | **OR 95% CI** | ***P*** |
| --- | --- | --- | --- | --- |
| Male | 171 | 66 (38.6%) | 0.634 (0.432–0.929) | 0.020 |
| Female | 245 | 131 (53.5%) | 0.713 (0.533–0.954) | 0.023 |
